# Effect of Er Miao San on peritoneal macrophage polarisation through the miRNA-33/NLRP3 signalling pathway in a rat model of adjuvant arthritis

**DOI:** 10.1080/13880209.2022.2066700

**Published:** 2022-05-24

**Authors:** Min Liu, Xiangwen Meng, Zihua Xuan, Simeng Chen, Jin Wang, Zhiluo Chen, Jiayu Wang, Xiaoyi Jia

**Affiliations:** a School of Pharmacy, Anhui University of Chinese Medicine, Hefei, China; b Anhui Province Key Laboratory of Chinese Medicinal Formula, Hefei, China; c Anhui Province Key Laboratory of Research & Development of Chinese Medicine, Hefei, China

**Keywords:** NLR family, pyrin domain-containing 3 protein, rheumatoid arthritis, anti-inflammatory agents, inflammasomes

## Abstract

**Context:**

Er Miao San (EMS) is a formulation that contains *Atractylodis Rhizoma* and *Phellodendri Cortex* in 1:1 ratio, and is commonly used to treat rheumatoid arthritis (RA) and other inflammatory diseases.

**Objective:**

We investigated the mechanism of action and effects of EMS on peritoneal macrophage differentiation in a rat model of adjuvant arthritis (AA).

**Materials and methods:**

EMS (3, 1.5 and 0.75 g/kg; once daily) and methotrexate (0.5 mg/kg; once every 3 days) were administered orally from days 21 to 35 after immunisation. Paw swelling and arthritis index were measured; pathological changes in the ankle joint were observed using x-ray and haematoxylin eosin staining. The ratio of CD86/CD206 in macrophages was detected by flow cytometry. Examination of the miRNA-33/NLRP3 signalling pathway was examined by RT-qPCR and western blotting. The levels of cytokines in the serum and cell supernatants were tested by ELISA.

**Results:**

EMS significantly reduced the AA index in rats (from 11.0 to 9.3) and pathological changes in the ankle joint (from 3.8 to 1.4). The ratio of CD86/CD206 was reduced, and polarisation to M1 improved (from 0.9 to 0.6) in macrophages of EMS-treated rats. EMS downregulated the miRNA-33/NLRP3 pathway. Furthermore, EMS treatment increased IL-10 and TGF-β levels in the serum and supernatant of macrophages of AA rats and simultaneously decreased the levels of IL-1β and TNF-α.

**Discussion and conclusions:**

Our results suggest that EMS may reduce macrophage polarisation to the M1 inflammatory phenotype by downregulating the miRNA-33/NLRP3 pathway in AA rats. These findings may provide new insights into the treatment of RA.

## Introduction

Rheumatoid arthritis (RA) is an autoimmune disease dominated by arthropathy, which affects ∼1%–2% of the population (Vande Walle et al. 2014). The main clinical manifestations are swelling and pain caused by synovial hyperplasia in the joint, followed by cartilage destruction, joint space narrowing and, at later stages, severe bone destruction and absorption, leading to joint stiffness, deformity and dysfunction. The pathogenesis of RA is complex, and its aetiology remains unclear. The abnormal proliferation of fibroblast synovial cells and excessive activation of immune cells, such as macrophages and dendritic cells may be the cause of RA (Hu et al. 2019). Two main subsets of polarized macrophages are found in the RA microenvironment: one is the classical activation type (M1), which mainly secretes pro-inflammatory cytokines, such as tumour necrosis factor (TNF)-α and interleukin (IL)-1β, causing joint erosion and the other is the replacement activation type (M2), which produces a large number of anti-inflammatory cytokines, such as IL-10 and tumour growth factor (TGF)-β, reduces local inflammation and promotes tissue healing and repair (Wang et al. 2017; Mohammadi et al. 2019). Multiple clinical studies have proven that the proportion of macrophages in patients with RA is unbalanced (Tardito et al. 2019), and that a large number of inflammatory macrophages infiltrate the synovial tissue (Soler Palacios et al. 2015). Therefore, suppression of macrophage-mediated inflammation may be a potential strategy for RA treatment.

MicroRNAs (miRNAs) are evolutionarily conserved endogenous non-coding RNA molecules consisting of 21–25 nucleotides that negatively regulate mRNA transcription and translation (Cheng et al. 2015). The miRNA profiles are different before and after RA, and they regulate many genes involved in the regulation of the NLRP3 inflammasome. miRNA-33, which is encoded in the introns of the sterol regulatory element-binding protein gene, plays an important role in inflammation (Lai et al. 2016), metabolism (Naar 2018) and mitochondrial function. miRNA-33 participates in the polarisation of inflammatory macrophages as a positive regulator of NLRP3 (Xie et al. 2018). NLRP3 inflammasome corpuscles are polyprotein complexes that belong to the nucleotide oligomerisation domain-like receptor family, and are closely associated with many inflammatory diseases. In the presence of exogenous pathogen-associated molecular patterns (PAMPs) and endogenous damage-associated molecular patterns (DAMPs), NLRP3 binds to pro-caspase-1 via the apoptosis-associated speck-like protein containing a C-terminal caspase recruitment domain (ASC) and activates the NLRP3 inflammasome. NLRP3 can induce pro-caspase-1 self-cleavage and activation, mediate the maturation and secretion of IL-1β and IL-18, and promote the inflammatory reaction (Long et al. 2020). Previous reports showed that the expression level of NLRP3 inflammasome corpuscle-associated protein in the synovial fluid and peripheral blood of patients with acute RA was significantly increased (Choulaki et al. 2015). Furthermore, the activation of the NLRP3 inflammasome alters the polarisation of M1 and M2 type macrophages (Zhang et al. 2018). However, it is not clear whether miRNA-33 regulates the activation of NLRP3 inflammasome corpuscles that promote macrophage polarisation to the M1 type and thus participate in the inflammatory response of RA.

Er Miao San (EMS), from the Yuan dynasty physician Zhu Danxi’s ‘Dan Xi Xin FA’, is a formulation that contains *Atractylodis Rhizoma* and *Phellodendri Cortex* in 1:1 ratio. *Phellodendri Cortex* is the dry bark of *Phellodendron Chinense* Schneid (Rutaceae); and *Atractylodis Rhizoma* is the dry rhizome of *Atractylodes lancea* (Thunb.) DC (Compositae). In modern clinical practice, EMS is commonly used in the treatment of gout, RA and other inflammatory diseases. In our previous study, We found that the ethyl acetate part of EMS could effectively inhibit arthritis and improve joint pathology in rats with adjuvant arthritis (AA); further, ultra-high performance liquid chromatography analysis showed that berberine and atractylodin may be responsible for the antiarthritic activity of the ethyl acetate part of EMS (Dai et al. 2020; Zhang et al. 2020). However, the mechanism of action of the ethyl acetate part of EMS in RA remains unclear. Herein, we investigate the potential molecular mechanisms underlying antiarthritic effects of the ethyl acetate part of EMS.

## Materials and methods

### Drugs

The Chinese herbs *Atractylodis Rhizoma* (1902120322) and *Phellodendri Cortex* (1901200062) were obtained from Bozhou (Anhui, China), identified by Dr. Liu SJ (School of Pharmacy, Anhui University of Chinese Medicine) and preserved in the Herbarium of School of Pharmacy, Anhui University of Traditional Chinese Medicine (Hefei, China; ID: EMS-19-01). Methotrexate (MTX) was obtained from the Xinyi Medical Limited Company (Shanghai, China).

### Preparation of the ethyl acetate fraction of EMS

The mixtures of the *Atractylodis rhizoma*, and *Phellodendri cortex* were dried, crushed and extracted thrice with water; the extraction times were 1.5, 1 and 0.5 h. The extract was evaporated to 500–600 mL at 60 °C. After five extractions with an equal volume of petroleum ether and ethyl acetate each, the ethyl acetate part of EMS was concentrated to pre-established doses (0.3, 0.15 and 0.075 g/mL; calculated according to the crude drug).

### Experimental animals and design

Specific Pathogen Free Sprague-Dawley (SD) rats (male, weight 160–180 g) were obtained from the Animal Department of Anhui Medical University (Hefei, China). The rats were placed in a standard laboratory (temperature controlled at 22 °C–26 °C, 12 h light/dark) and allowed to drink and eat freely. The Experimental Animal Ethics Committee of Anhui University of Traditional Chinese Medicine approved all experiments (No.: 2020016).

Inactivated Bacillus Calmette-Guerin vaccine (80 °C, 1 h) was fully mixed with liquid paraffin and sterilized at by high pressure. Complete Freund’s adjuvant (CFA) was prepared at 10 mg/mL concentration and then 0.1 mL of CFA was injected into the right hind foot of rats, whereas a normal control group was injected with normal saline. On the 20th day after immunisation, the rats were randomly divided into normal, AA model, EMS (3, 1.5 and 0.75 g/kg) and MTX (0.5 mg/kg) groups, according to the systemic manifestations and arthritis index score. EMS was administered intercostally once daily for 14 days, while MTX group was administered intercostally once every three days. Simultaneously, the normal and AA model groups were administered an equal amount of carboxymethyl cellulose solution (10 mL/kg).

### Evaluation of arthritis

Electronic scales were used to record the weight of the rats every 7 days. Hind paw volume was measured with a PV-200 volume metre (Chengdu Technology Market, Chengdu, China) before (base value) and after immunisation (days 20, 23, 26, 29, 32 and 35). AA rats were immunized at different time points to determine the polyarthritis index. The degree of paw and ankle swelling was evaluated from 0 to 4 points, with a maximum score of 16 for each rat limb (Chyuan et al. 2018).

### Ankle x-ray imaging and histopathology

Rats were sacrificed 14 days after administration. The left posterior ankle joints were collected and the surrounding muscles, tendons and other soft tissues were removed and fixed with 4% paraformaldehyde, part of which was used to observe whether the ankle joint was deformed. The other parts were decalcified with 5% HNO_3_, embedded in paraffin, sectioned, stained with haematoxylin eosin (HE) and the histopathological changes in each group were observed using an optical microscope. Synovial cell proliferation, cell infiltration, pannus formation and bone erosion were classified into four grades according to the degree of severity: grade 0, no significant change; grade 1, mild; grade 2, moderate; and grade 3, severe.

### Culture of peritoneal macrophages (PMs)

After the rats were sacrificed, the abdomens were swabbed with alcohol cotton balls. The PMs were collected by injecting 10 mL of sterilized phosphate-buffered saline (PBS) into the abdominal cavity, gently massaging for 5 min and then resting for 5 min. The cell suspension was extracted and centrifuged at 2500 rpm (Thermo Scientific, USA) for 10 min; then, 10% Dulbecco’s Modified Eagle Medium (DMEM) was added to resuspend the cell pellet. The cells were seeded into a six-well plate at a density of 1 × 10^6^ cells/mL and incubated in an incubator at 37 °C and 5% CO_2_ for 4 h. The supernatant was discarded and the cells were washed thrice with sterile PBS to obtain the adherent purified macrophages.

### Flow cytometry

Cells (1 × 10^6^) were collected and centrifuged at 2500 rpm for 10 min to prepare the cell suspensions. Then, fluorescently labelled antibody CD86-APC (Miltenyi Biotec, Germany) was added and the cells were incubated for 30 min in the dark. After washing, the cell membrane was fixed and permeated and fluorescently labelled antibodies CD68-Fluorescein isothiocyanate (FITC; Miltenyi Biotec, Germany) and CD206-PE (Santa Cruz, CA, USA) were added, and the cells were again incubated for 30 min in the dark. The suspension was washed once with PBS and the cells were resuspended in 300 μL PBS, filtered and analysed.

For phagocytosis evaluation, FITC dextran (Sigma, St. Louis, MO, USA) was dissolved in sterile water to obtain mother liquor (25 mg/mL). The mother liquor (40 μL) was dissolved in 960 μL of DMEM containing 10% Foetal Bovine Serum to prepare the working liquid. The cells were collected and centrifuged at 2500 rpm for 10 min. The working fluid (1 mL) was added into each tube and was prepared according to the sample quantity. The samples were incubated in dark for 1.5 h. Another tube was prepared, as a negative control and incubated at 4 °C for 1.5 h. After 1.5 h, the cells were collected, centrifuged at 2500 rpm for 10 min to discard the supernatant, resuspended in 300 μL of PBS, filtered and analysed using a flow cytometer (FC500, Beckman, Brea, CA, USA).

### Enzyme-linked immunosorbent assay (ELISA)

The rat serum and PM culture cell supernatant were collected, and the expression levels of IL-10, TGF-β, IL-1β and TNF-α (Shanghai Jianglai Biotechnology, China) were detected by ELISA according to the manufacturer’s instructions.

### Real-time-quantitative polymerase chain reaction (RT-qPCR)

The PMs were treated with Trizol reagent (Thermo Fisher, Waltham, MA, USA) for total RNA extraction. The ReverTra Ace q-PCRT Master Mix (TOYOBO, Japan) kit was used to reverse transcribe RNA into cDNA, and the SYBR Green Realtime PCR Master kit (TOYOBO, Japan) was used to quantify the mRNA content using the PCR instrument (Stratagene, San Diego, CA, USA, MX3000P) according to the manufacturer’s instructions. miRNA was quantified using the All-in-One TM miRNA Q-PCR Detection System (GeneCopoeia, Rockville, MD, USA). Relative gene expression levels were calculated using the 2^–ΔΔCt^ method. The primers used were listed as follows: NLRP3 (forward: 5′-CAGACCTCCAAGACCACGACTG-3′; reverse: 5′-CATCCGCAGCCAATGAACAGAG-3′), ASC (forward: 5′-TTATGGAAGAGTCTGGAGCTGTGG-3′; reverse: 5′-AATGAGTGCTTGCCTGTGTTGG-3′), caspase-1 (forward: 5′-TGCCTGGTCTTGTGACTTGGAG-3′; reverse: 5′-ATGTCCTGGGAAGAGGTAGAAACG-3′), β-actin (forward: 5′-GGAGATTACTGCCCTGGCTCCTA-3′; reverse: 5′-GACTCATCGTACTCCTGCTTGCTG-3′), miRNA-33 (5′-TGCGCGTGCATTGTAGTTGCATTGCA-3′). The internal reference was U6.

### Western blot analysis

After cell collection and total protein extraction, the proteins were separated by sodium dodecyl sulphate-polyacrylamide gel electrophoresis and then transferred onto poly vinylidene fluoride membranes. The membranes were blocked with 5% skim milk at room temperature for 2 h, washed three times with tris-buffered saline Tween (TBS-T) solution and incubated with a primary antibody at 4 °C overnight. Subsequently, the membranes were incubated with anti-NLRP3 (1:1000 Abcam, Cambridge, MA, USA), ASC (1:1000 Affinity, America), caspase-1 (1:1000 Affinity, America), P-caspase-1 (1:1000 Abcam, MA, USA), iNOS and Arg-1 (1:1000 Zen BioScience, China) primary antibodies. The membranes were washed thrice with TBS-T and incubated with horseradish peroxidase-conjugated secondary antibodies (Sparkjade, China) for 2 h at room temperature. After subsequent washing with TBS-T solution thrice, protein bands were detected using an ECL (Affinity, America) kit. Finally, the band intensities were quantitatively analysed using ImageJ analysis software.

### Statistical analysis

The average values of the experimental data ± standard deviation were calculated. SPSS software was used to analyse the data using one-way analysis of variance. The *p* < 0.05 was considered to be statistically significant.

## Results

### Effects of EMS on the overall rat AA index

**Figure 1.**
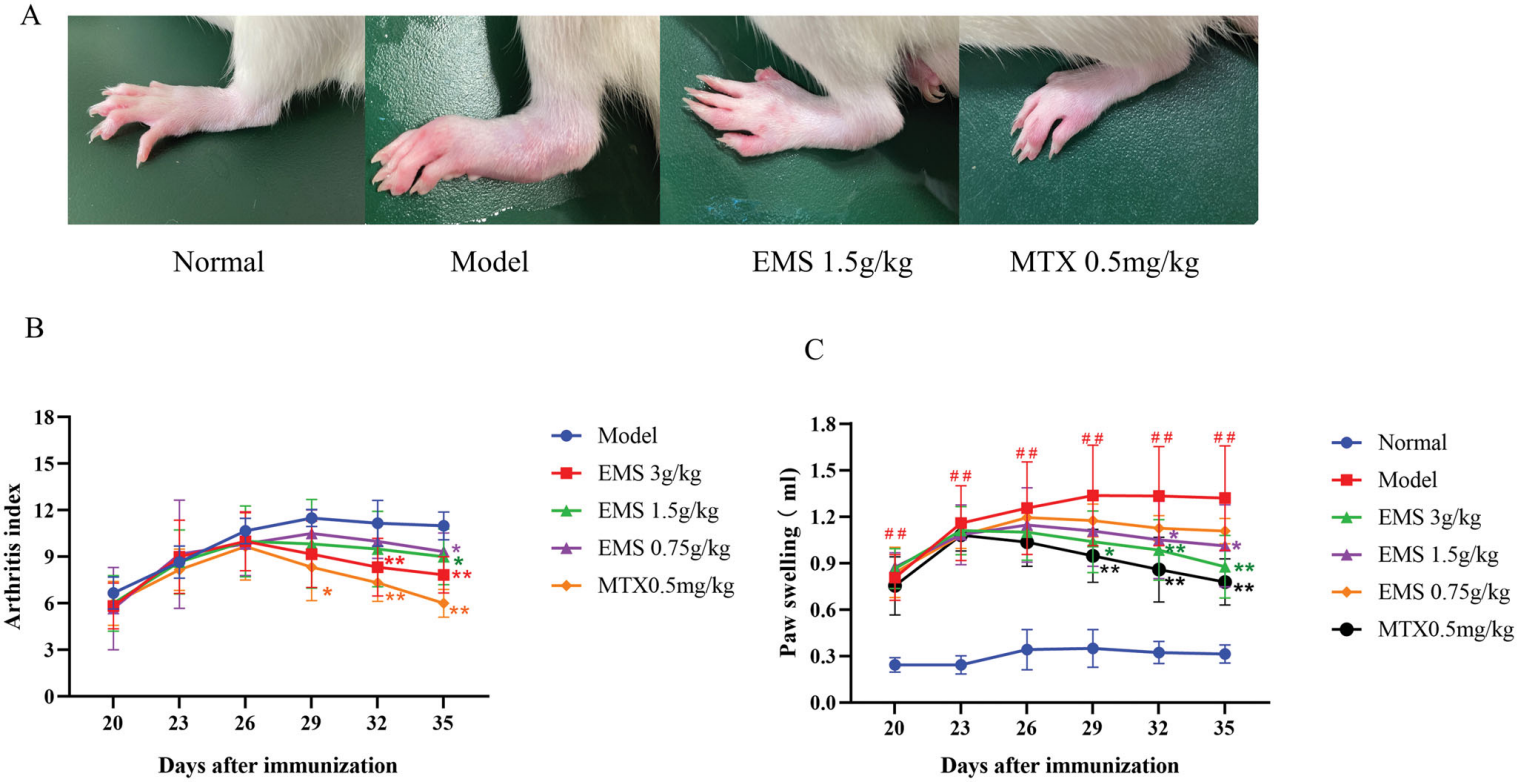
The effects of EMS on the overall index of AA rats. (A) The effects of EMS on the paws of AA rats (day 35). (B) The effects of EMS on paw swelling and (C) polyarthritis index in AA rats. ##*p* < 0.01 vs. the normal group; *p < 0.05; **p < 0.01 vs. the model group (*n* = 6).

As shown in Figure 1(A), the AA rats had severe swelling in the side paws and multiple joint nodules. After EMS and MTX treatments, the paw swelling reduced. As shown in Figure 1(B,C), we found that a secondary inflammatory reaction appeared around days 20–23 and peaked around days 26–29. The EMS significantly reduced paw swelling (from 1.4 to 0.9, *p* < 0.01) and arthritis index (from 12 to 6, *p* < 0.01) compared to the AA model group. These results indicated that EMS could effectively treat AA in rats.

### Effects of EMS on AA rat ankle joint pathology

**Figure 2.**
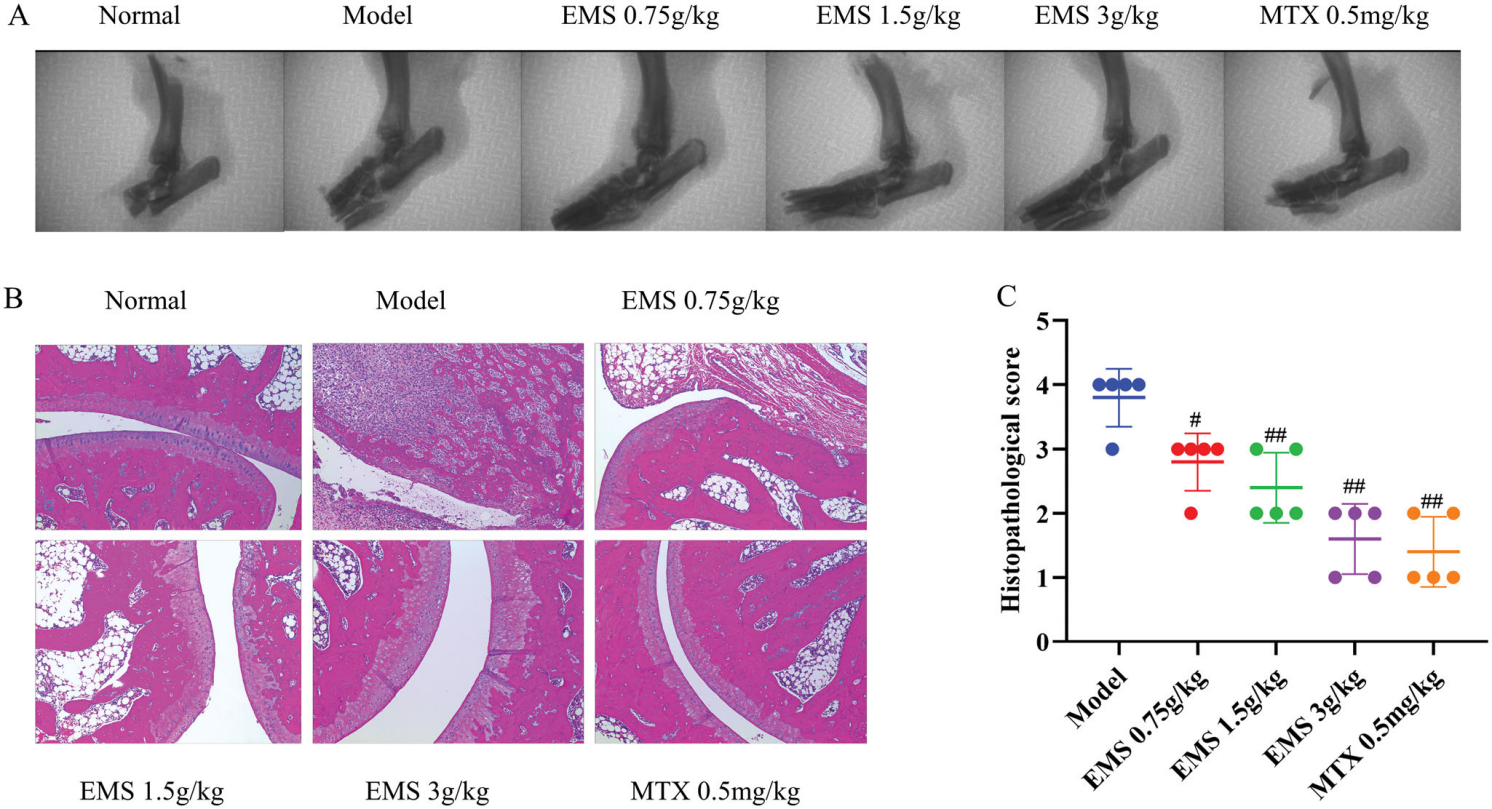
X-ray imaging and HE staining for the evaluation of the effects of EMS on AA large ankle joint lesions. (A) X-ray image of ankle joint of AA rats (related parameters: image resolution 15 LP/min; photography conditions 22.0 KV, 4 mA, 0.1 mGy); (B) representative images showing the effects of EMS on ankle pathology in AA rats (HE, ×100 magnification); (C) the histopathological scores of the rats. #*p* < 0.05; ##*p* < 0.01 vs. the model group (*n* = 5).

X-ray detection is considered a ‘gold standard’ for clinical diagnosis of RA, and histopathological analysis is an important indicator of clinical symptoms. Here, we used x-ray imaging and histopathological analysis to determine the antiarthritic effects of EMS. As shown in Figure 2(A), ankle joints of rats in the AA model group were deformed and fragmented, and the joint spaces were narrowed compared to those in the normal group. After treatment with EMS and MTX, joint injuries significantly improved. Histopathological analysis showed smooth joint surfaces in the normal group and abundant inflammatory cell infiltration, synovial hyperplasia, cartilage erosion and bone destruction in the AA model group. After EMS administration, the histopathological changes in the joints significantly, ameliorated while the severity scores of each group substantially decreased (Figure 2(C); *p* < 0.01). These results showed that EMS improved pathological conditions of the ankle joint and exerted a protective effect on AA rats.

### Effects of EMS on the expression of cytokines in AA rat serum

**Figure 3.**
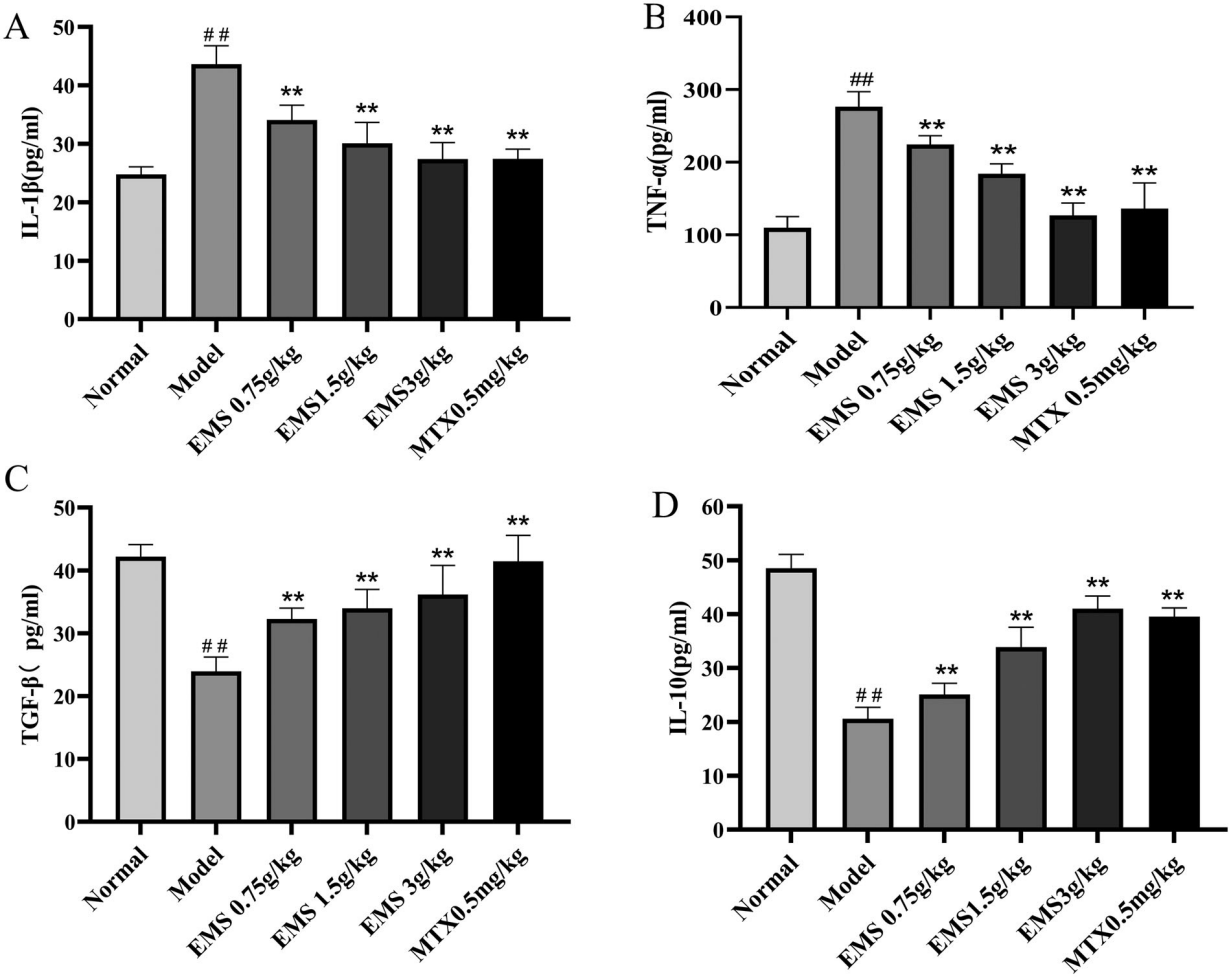
The effects of EMS on the production of serum cytokines in AA rats. The levels of TNF-α, IL-1β, TGF-β and IL-10 in serum samples were detected by ELISA. (A) IL-1β, (B) TNF-α, (C) TGF-β and (D) IL-10. ##*p* < 0.01, vs. the normal group; **p < 0.01 vs. the model group (*n* = 6).

Serum samples were collected from each group, and serum cytokine levels were detected by ELISA. As shown in Figure 3(A,B), the levels of TNF-α and IL-1β were increased in AA model group as compared with those in the normal group (*p* < 0.01), moreover, the levels of TGF-β and IL-10 decreased (*p* < 0.01; Figure 3(C,D)). Treatment with EMS (0.75, 1.5 and 3 g/kg) and MTX (0.5 mg/kg) significantly reduced the levels of TNF-α and IL-1β (Figure 3(A,B)), but increased those of TGF-β and IL-10 (Figure 3(C,D)). These results suggest that EMS may regulate the levels of these cytokines in a dose-dependent manner, and that the ability of EMS to induce AA protection may involve restoring the balance between pro-inflammatory and anti-inflammatory cytokines.

### Effects of EMS on PM polarisation in AA rats

**Figure 4.**
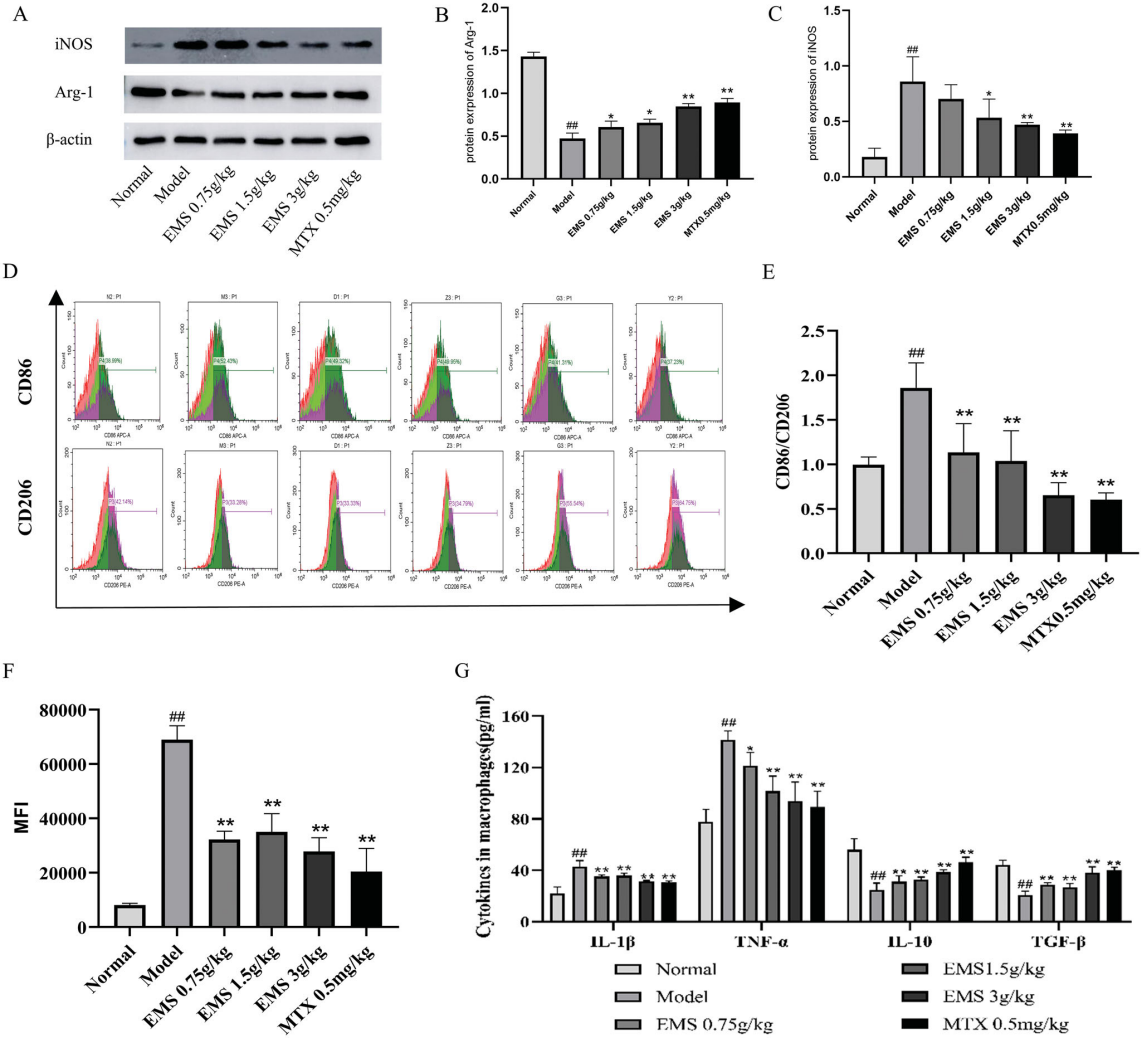
The effects of EMS on the expression of M1 and M2 marker proteins iNOS and Arg-1 in PMs of AA rats. (A) Western blotting was used to detect the expressions of iNOS and Arg-1 in PMs of AA rats. (B, C) Densitometric analyses of iNOS and Arg-1 normalized to histone β-actin. The effects of EMS on the CD86/CD206 ratio and phagocytic ability. (D) CD86, CD206 and phagocytic ability were detected by flow cytometry. (E) The ratio of CD86/CD206 in PMs of AA rats. (F) The mean fluorescence intensity of phagocytic ability was calculated. (G) The levels of TNF-α, IL-1β, TGF-β and IL-10 were detected by ELISA. ##*p* < 0.01 vs. the normal group; *p < 0.05; **p < 0.01 vs. the model group (*n* = 3).

To examine the role of EMS in the M1/M2 polarisation of PMs in AA rats, PMs were extracted and the ratio of CD86/CD206 was determined by flow cytometry. As shown in Figure 4(D,E), the ratio of CD86/CD206 was significantly higher in the PMs of AA model rats than in those of the normal group (from 1.0 to 1.8, *p* < 0.01). The EMS (0.75, 1.5, 3 g/kg) and MTX (0.5 mg/kg) treatment significantly reversed the ratio of CD86/CD206, reduced the expression of M1-labelled CD86 and induced the polarisation of macrophages to the M2 pro-inflammatory phenotype as compared with the model group. Western blot analysis further confirmed that EMS (3 g/kg) significantly decreased M1 marker iNOS expression level (*p* < 0.01) and increased the expression levels of Arg-1 marker protein (*p* < 0.01). EMS (0.75, 1.5 g/kg) also increased the expression of M2 marker Arg-1 (*p* < 0.05; Figure 4(A–C)).

In addition, we examined macrophage phagocytosis using flow cytometry (Figure 4(F)). With the development of inflammation, the PMs of AA model rats were abnormally activated, and their phagocytosis was abnormally enhanced (*p* < 0.01). the abnormal phagocytic ability of macrophages was significantly inhibited by EMS (0.75, 1.5 and 3 g/kg) and MTX (0.5 mg/kg). During the development of inflammation, the pro-inflammatory factors TNF-α and IL-1β are mainly secreted by M1 macrophages, while the anti-inflammatory factors TGF-β and IL-10 are primarily secreted by M2 macrophages. Here, we used ELISA to detect cytokines levels in the PMs of rats. The levels of TNF-α and IL-1β in AA rats were higher than in normal rats, whereas the levels of TGF-β and IL-10 were lower (Figure 4(G)). After treatment with EMS (0.75, 1.5 and 3 g/kg) and MTX (0.5 mg/kg), the levels of TNF-α and IL-1 β decreased, whereas the levels of TGF-β and IL-10 significantly increased (Figure 4(G)). These findings indicate that EMS may reduce the ratio of M1/M2 PMs in AA rats and regulate the balance between pro-inflammatory and anti-inflammatory cytokines secreted by M1 and M2 macrophages.

### Effects of EMS on miRNA-33/NLRP3 expression in the PMs of AA rats

**Figure 5.**
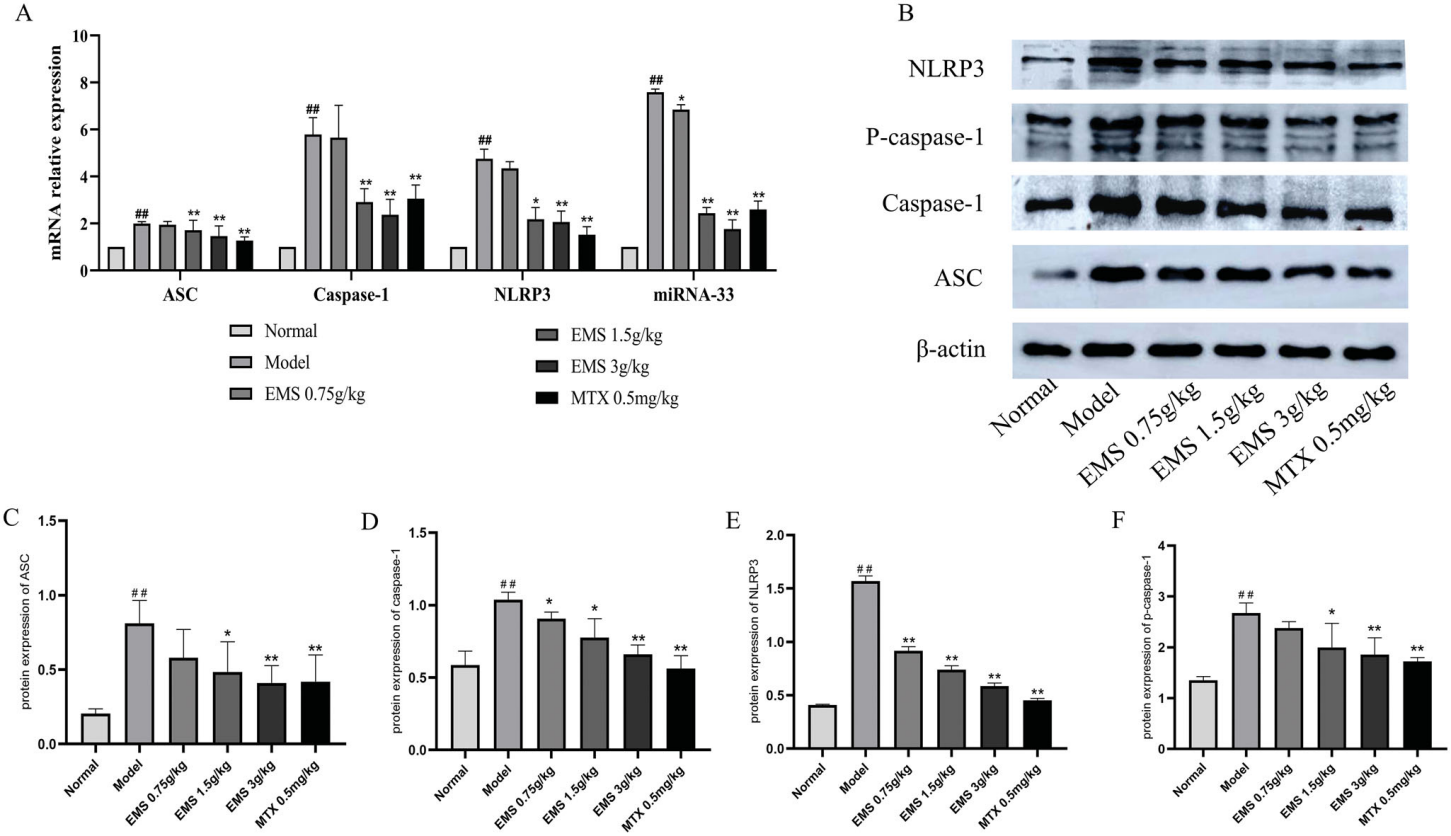
The effects of EMS on expression of miRNA-33/NLRP3 in the PMs of AA rats. miRNA-33, NLRP3, ASC and caspase-1 mRNAs were detected by RT-qPCR. (A) mRNA levels of miRNA-33, NLRP3, ASC and caspase-1. The effects of EMS on the expression of NLRP3 inflammasome-associated protein in the PMs of AA rats. (B) The protein levels of NLRP3, ASC, caspase-1, pro-caspase-1 were determined by western blotting. Densitometric analyses of the NLRP3 inflammasome-associated protein normalized to histone β-actin. (C) ASC, (D) caspase-1, (E) NLRP3 and (F) pro-caspase-1. ##*p* < 0.01, vs. the normal group; **p < 0.01 vs. the model group (*n* = 3).

To investigate the cause of macrophage polarisation, we further evaluated the expression of NLRP3 inflammatory corpuscles. As shown in Figure 5, EMS (0.75 and 1.5 g/kg) strongly inhibited the mRNA and protein expression levels of NLRP3 inflammatory corpuscles *in vivo*. miRNA-33 is reported to be a positive regulator of NLRP3 inflammatory bodies; therefore, we studied the effects of EMS on miRNA-33. RT-qPCR showed that EMS reduced the miRNA-33 levels. These results suggest that EMS may affect the polarisation of PMs to M1 by downregulating miRNA-33/NLRP3 in AA rats.

## Discussion

In this study, we reported the expression of miRNA-33/NLRP3 in the PMs of AA rats and determined the effects of EMS on macrophage polarisation. EMS plays an important role in regulating the miRNA-33/NLRP3 axis in macrophage polarisation. These findings reveal a possible mechanism for macrophage polarisation that affects the development of RA.

RA is a chronic and systemic autoimmune disease characterized by synovial joint inflammation. Its aetiology and pathogenesis are unclear, however, a high level of synovial tissue macrophage infiltration is considered to be an early indicator of RA (Haringman et al. 2005). Macrophages secrete a variety of pro-inflammatory factors that accelerate the development of RA. In several studies, the peripheral blood, synovial fluid and synovial tissue of patients with RA, and animal models of RA were found to have an imbalance in the number of M1 and M2 macrophages (Tardito et al. 2019; Yang et al. 2019; Zhou et al. 2019). At present, some new nano-preparations such as folic acid coated silver nanoparticles (Yang et al. 2021) and composites of micro-fibrillated cellulose with mesoporous silica nanoparticles (Kim et al. 2019) can be actively transported to M1 macrophages, and can synergistically reduce macrophage M1 and M2 polarisation to effectively treat animal models of RA. These findings indicate that macrophage polarisation affects the occurrence and development of RA. Inducing macrophages to polarize to the M2 phenotype, maintaining the balance between M1 and M2 macrophages and preventing the occurrence and development of inflammation may be a breakthrough in the treatment of RA. In the present study, we investigated the effects of the EMS on the polarisation of macrophages, and found that EMS reduced the CD86/CD206 ratio in the PMs of AA rats, altered the expression of macrophage M1/M2 marker proteins, regulated the balance between anti-inflammatory and pro-inflammatory factors and reduced joint inflammation and foot swelling.

The activation of the NLRP3 inflammasome is an important factor in the development of RA. Under the action of exogenous PAMPs and endogenous DAMPs, NLRP3 binds to pro-caspase-1, through the adaptor protein ASC, to activate the NLRP3 inflammasome. After activation of NLRP3, pro-caspase-1 can self-cleavage formation of mature caspase-1, thus inducing the maturation and secretion of IL-1β (Kim and Jo 2013). IL-1β plays a major role in cell inflammation (Choulaki et al. 2015). The inhibition of NLRP3 inflammation can transform Lipopolysaccharide (LPS)-stimulated M1 macrophages into the M2 phenotype (Zhang et al. 2018). Therefore, after evaluating the effects of the EMS on AA model rats by changing the ratio of M1/M2 macrophages, we investigated the expression of the NLRP3 inflammasome and found that EMS inhibited the mRNA and protein expression of NLRP3. This finding suggests that EMS reduces the polarisation of macrophages to M1 and induce the polarisation to M2 by inhibiting the NLRP3 inflammasome, thus leading to the secretion of more anti-inflammatory factors and improving RA symptoms.

Recent studies have shown that miRNAs also controls many genes involved in NLRP3 regulation and affect the development of RA (Park et al. 2015; Tang et al. 2019). miRNA-33 induces mitochondrial dysfunction and triggers the activation of NLRP3 in LPS-induced mouse macrophages (Xie et al. 2018). Moreover, miRNA-33 can activate NLRP3 inflammation through Ogg-1, which aggravates the development of lipid inflammation (Tumurkhuu et al. 2016). In addition, the level of miRNA-33 in peripheral blood mononuclear cells was found to be significantly higher in patients with RA, than in the healthy control group. Therefore, we hypothesized that the ethyl acetate of EMS may affect the polarisation of PMs in AA rats via the miRNA-33/NLRP3 signalling pathway. Our results showed that the expression of miRNA-33 in the PMs of AA rats increased notably, and that EMS can reduce the expression levels of miRNA-33 and NLRP3. These results indicate that EMS may reduce the polarisation of macrophages to M1 through the miRNA-33/NLRP3 signalling pathway to exert anti-inflammatory effects. Further, our results suggest a potential role of the miRNA-33/NLRP3 signalling pathway in RA. On the contrary, showed that miRNA-33 inhibited the release of CCL2 in the supernatant of primary mouse chondrocytes, which could recruit monocytes to infiltrate joint tissue and aggravate the development of osteoarthritis (Wei et al. 2016). These findings suggest that the miRNA-33/NLRP3 pathway may not exist in chondrocytes, or that its function may be antagonized by other miRNA-33 promoters. The most important limitation of this study lies in the fact that the relationship between the miRNA-33/NLRP3 pathway and RA was explored only in animals. Therefore, the mechanisms involved in the miRNA-33/NLRP3 signalling pathway in macrophages and other cells should be further investigated *in vitro*.

## Conclusions

Our results showed that EMS reduced the polarisation of PMs to the M1 phenotype in AA rats and induced their polarisation to the M2 phenotype, thus reducing the secretion of pro-inflammatory factors and producing more anti-inflammatory factors to regulate the balance of cytokines, thereby, exerting a protective effect on AA rats. This effect may be mediated through the miRNA-33/NLRP3 signalling pathway.
